# A metabolomics and proteomics study of the *Lactobacillus plantarum* in the grass carp fermentation

**DOI:** 10.1186/s12866-018-1354-x

**Published:** 2018-12-18

**Authors:** Tinghong Ming, Jiaojiao Han, Yanyan Li, Chenyang Lu, Dihong Qiu, Ye Li, Jun Zhou, Xiurong Su

**Affiliations:** 1 College of Food and Pharmaceutical Sciences, Ningbo University, 169 Qixing South Road, Meishan, Ningbo, China; 2 Department of Food Science, Cornell University, New York, USA; 3 School of Marine Sciences, Ningbo University, 818 Fenghua Road, Ningbo, China; 4 Hangzhou Medical College, Hangzhou, China

**Keywords:** *Lactobacillus plantarum*, Grass carp, Fermentation, Metabolomics, Proteomics

## Abstract

**Background:**

*Lactobacillus plantarum*, a versatile lactic acid-fermenting bacterium, isolated from the traditional pickles in Ningbo of China, was chosen for grass carp fermentation, which could also improve the flavor of grass carp. We here explored the central metabolic pathways of *L. plantarum* by using metabolomic approach, and further proved the potential for metabolomics combined with proteomics approaches for the basic research on the changes of metabolites and the corresponding fermentation mechanism of *L. plantarum* fermentation.

**Results:**

This study provides a cellular material footprinting of more than 77 metabolites and 27 proteins in *L. plantarum* during the grass carp fermentation. Compared to control group, cells displayed higher levels of proteins associated with glycolysis and nucleotide synthesis, whereas increased levels of serine, ornithine, aspartic acid, 2-piperidinecarboxylic acid, and fumarate, along with decreased levels of alanine, glycine, threonine, tryptophan, and lysine.

**Conclusions:**

Our results may provide a deeper understanding of *L. plantarum* fermentation mechanism based on metabolomics and proteomic analysis and facilitate future investigations into the characterization of *L. plantarum* during the grass carp fermentation.

**Electronic supplementary material:**

The online version of this article (10.1186/s12866-018-1354-x) contains supplementary material, which is available to authorized users.

## Background

*Lactobacillus plantarum*, a facultative anaerobic homo-fermentative bacterium, is a widespread and well-documented lactic acid bacterium (LAB) species. *L. plantarum* have been evaluated for their industrial fermentation potential, including spoilage-preventing and preservative for raw-materials, as well as its application for improving flavor and texture of fermented product [1, 2]. *L. plantarum*, as an auspicious natural starter in industrial fermentation, can enhance the formation of the desired metabolites and flavor profiles, and improve the taste of fermented end-products [3].

Grass carp (*Ctenopharyngodon idellus*) is one of the most important farmed freshwater fish species in China, which assembles in its easy cultivation, fast growth rate, high nutritional value and low price. The potential of this fish, as one kind of low fat and high protein-containing food, have not yet been fully utilized because of unpleasant fishy odour and limited processing, as well as the storage period and distribution sphere [4]. It was surveyed that only a very small percentage of grass carp caught in China were processed, mainly into traditional salted fish and smoked fish products [5, 6]. Generally, traditional grass carp fermentation occurs during the processing of salted fish products by a commensal group of microrganism [7]. However, in recent years, with increasing concerns about the safety of salted and smoked fish, the interest in fermenting grass carp has been of increasing concern for researchers. Liu et al. used the mixed starters (*Lactobacillus casei*, *Streptococcus lactis*, *Saccharomyces cerevisiae* Hansen and *Monascus anka*) to ferment grass carp muscles, and the result showed that this technique could significantly improve the quality of processed grass carp, and control the accumulation of biogenic amine [8]. Moreover, studies had shown that grass carp fermentation using LAB could not only improve the flavor and sensory qualities of the product, but could also extend the shelf life of fermented product [9]. However, the metabolite profile changes during the grass carp fermentation by *L. plantarum* remain unclear.

Metabolite profiling, aimed to monitor all metabolites in a biological sample, had been used to evaluate the changes of metabolites, which could be considered as the ultimate responses of biological systems to the environmental variations [10]. So far, several high-throughput analytical techniques have been used for metabolite profiling [11]. Among these techniques, gas chromatography-mass spectrometry (GC-MS) can provide a relatively high reproducibility, good sensitivity, high resolution, and high-throughput analysis, which can be used for analyzing the primary metabolism products, including amino acids, organic acids, carbohydrates and fatty acids [12]. However, most of these metabolites are nonvolatile, thus derivatization must be carried out prior to the analysis by GC-MS [13]. Currently, the most versatile and universally applicable derivatization technique is silylation, which can derivatize compounds containing polar functional groups by adding a trimethylsilyl (TMS) reagent to form TMS compounds [14]. *N*,*O*-bis (trimethylsilyl) trifluoroacetamide with 1% trimethylchlorosilane is commonly used as silylation reagent and derivatization method with GC-MS, which has been widely used for the analysis of various biological metabolic footprinting [15, 16]. For instance, Johanningsmeier et al. determined 92 metabolites of *Lactobacillus buchneri* strain LA1147 during anaerobic spoilage of fermented cucumbers by this derivatization method [17]. Additionally, there have also been some researches on the catalyzing compound pyridine that can improve the silylation power of silylation reagents [14]. Considering the derivatization-based GC-MS has been reported for the analysis of metabolites in microbial sample, thus GC-MS can be used to identified the changes of *L. plantarum* intracellular metabolites.

Proteomics is a subject that explores the existence and activity patterns of specific proteins in different time and space in cells [18], which is involved in qualitative, quantitative, localization, modification, activity, function, interaction and differential expression of protein etc., to explore the proteins of expression and functional pattern from the protein level [19]. Two-dimensional electrophoresis (2-DE) as a powerful analytical tool can be used for the study of protein expression profiles to acquire protein differential expression information and illuminate molecular mechanisms, such as the mechanism of probiotic bacteria for fermentation [20]. Currently, some progress has been achieved in the proteomics research relating to LAB total proteins and environmental stress or growth conditions. For example, a proteomic analysis profiled the proteins of *L. plantarum* strains under food-like conditions [21].

In the current study, a combined metabolomics and proteomics approach was employed to illuminate the metabolites and protein changes during the grass carp fermentation by *L. plantarum.*

## Results

### Metabolomic profiling through GC- MS

**Fig. 1:**
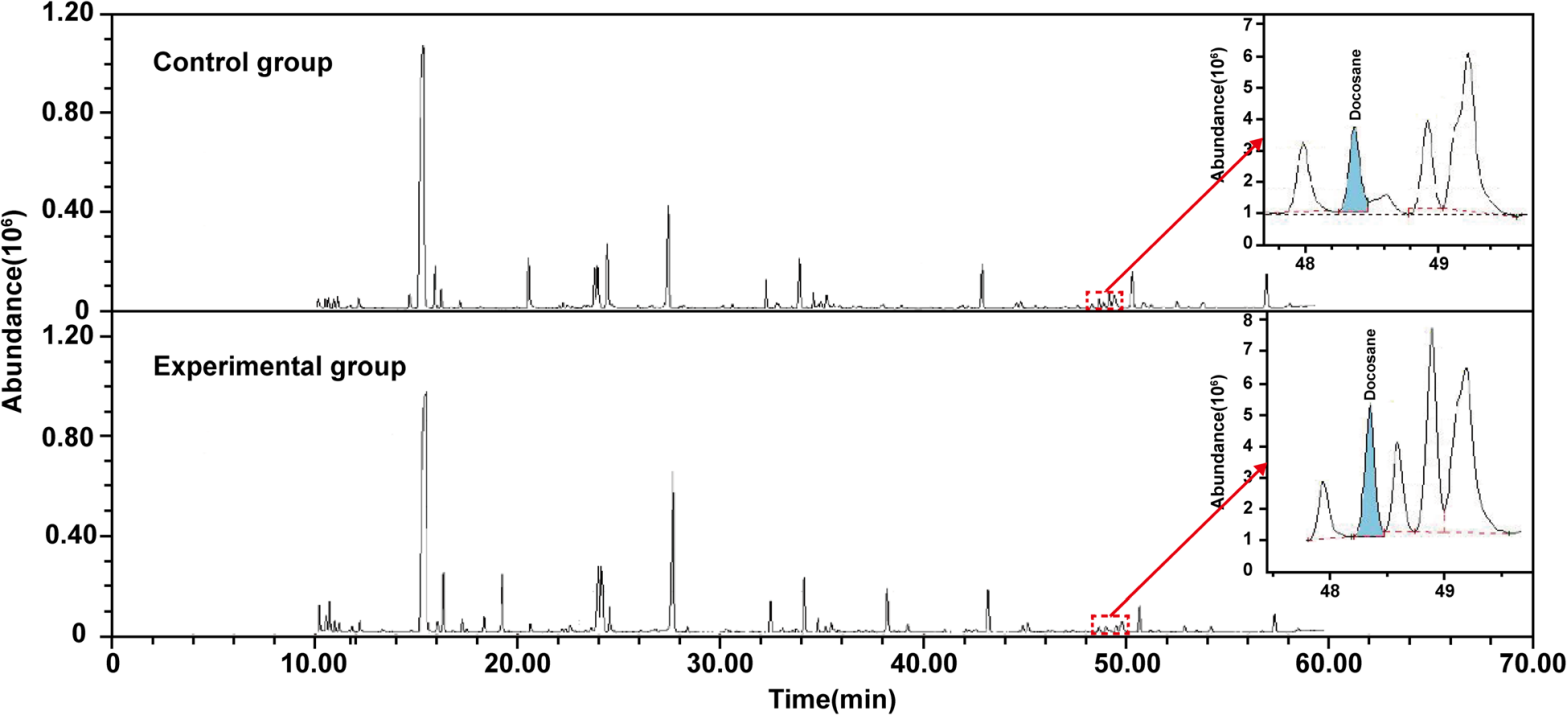
The typical total ion chromatograms (TICs) of *Lactobacillus plantarum* intracellular metabolites. The internal standard sample was shown in red dotted box

Metabolic profiles for the typical GC-MS total ion chromatograms (TICs) between the control and experimental groups are illustrated in Fig. 1. According to the TICs, a total of 90 endogenous metabolites (including eight uncertain metabolites) were identified in *L. plantarum*, including 74 differential metabolites (Additional file 1: Table S2). Then, the concentrations of 74 and 77 metabolites in the control and experimental groups were calculated based on the internal standard peak area in Additional file 1: Table S2.

**Fig. 2:**
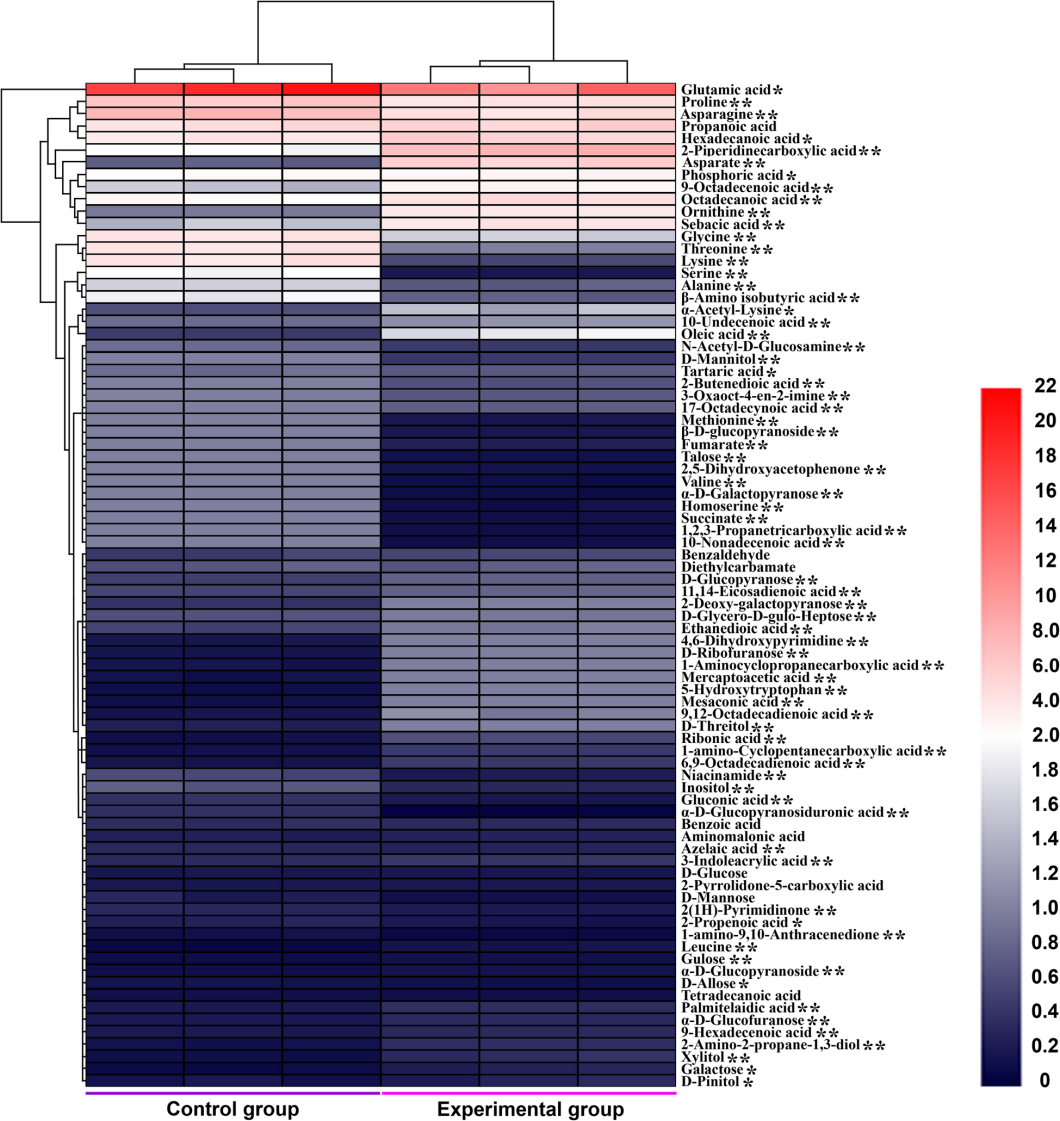
Hierarchical cluster analysis for differentially identified metabolites in the control group and experimental groups. Each column and row respectively represent a measurement group and an individual metabolite. The colors were according to the averages of relative concentration of each sample. Values with * are significantly different from the control group. ^*^p < 0.05, ^**^p < 0.01

As shown in Additional file 2: Figure S1, 82 metabolites between control and experimental group were used to build a PCA model and loadings plot. The scores plot from PCA presented clear discrimination between the intracellular metabolome of two groups. Further, hierarchical cluster analysis of resulting 82 identified metabolites revealed the existence of distinct differences between the control and experimental groups (Fig. 2). According to the clustering result of metabolites, serine, glycine, threonine, proline, asparagine, valine, glutamic acid, propanoic acid, lysine, hexadecanoic acid, octadecanoic acid and ornithine were abundant in each sample.

### Differentially expressed proteins (DEPs)

**Fig. 3:**
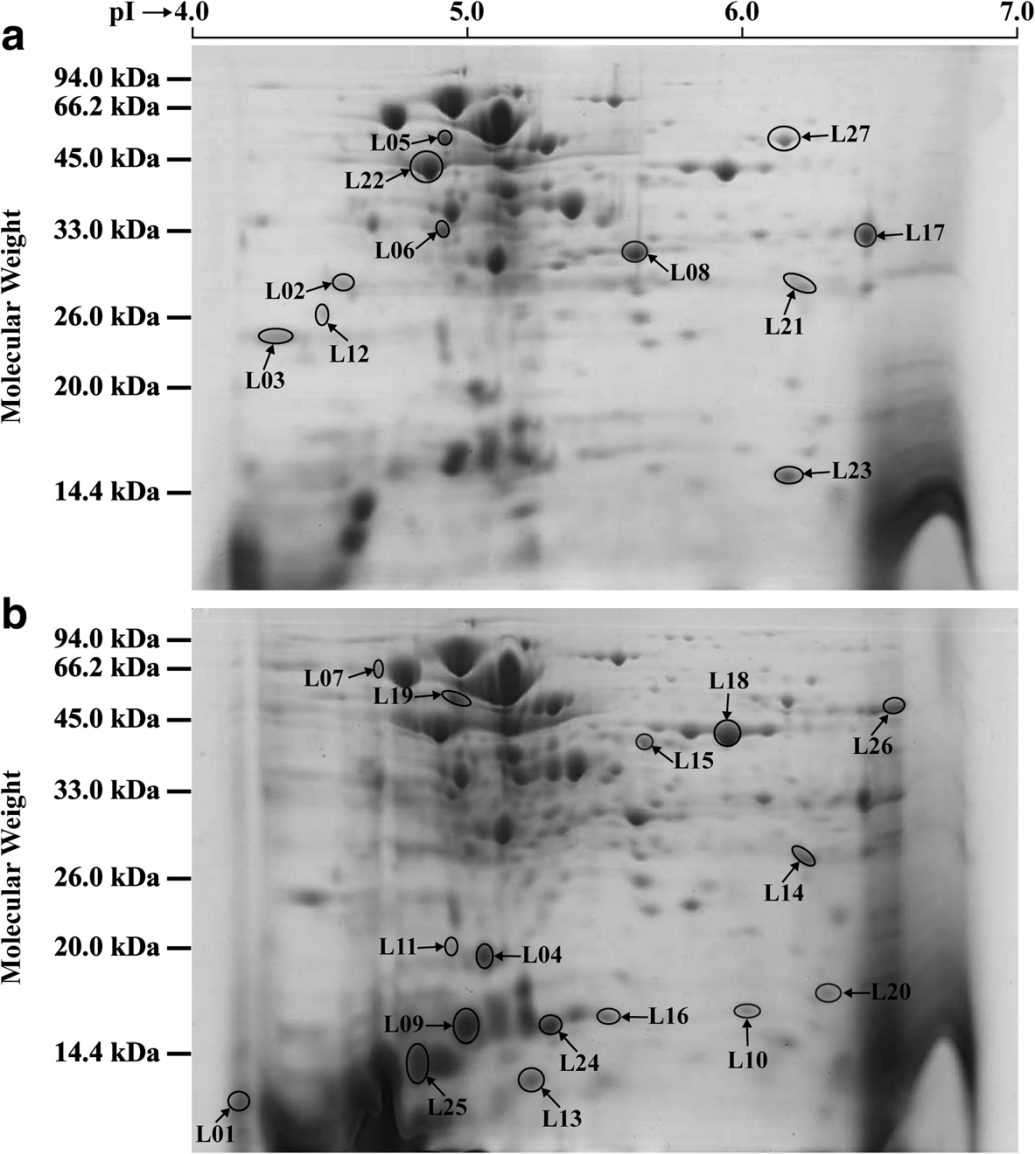
2-DE map of the proteome extracted from *Lactobacillus plantarum* cells. **a** control group, **b** experimental group. The change of spots mean intensity is illustrated in the different 2-DE maps, and arrows with their respective number have been labeled in the selected for MS/MS identification

Proteins composed of 260 ± 22 and 299 ± 25 spots between the control and experimental groups, and coupled with 191 ± 21 spots in common, were presented in Fig. 3. These spots were mainly distributed in the pI range of 4–7 and molecular mass of 14–94 kDa. After three biological replicates of 2-DE gels were performed by background subtraction, normalization and spot match, the relative volume of each spot was determined by its spot intensity in pixel units. Then, the protein spot was normalized to the sum of the intensities of all the spots of the gel to calculate the vol% (percent by volume) [22]. If the mean normalized spot volume was varied at least 1.5-fold compared to the control spots, combined with the analysis of variance at a significance level of *p* < 0.05, this protein was considered as the DEP [23]. Here, the DEPs spots with 1.5-fold change and *p* < 0.05 were analyzed by PDQuest software, as depicted in Fig. 3. In total, 11 and 16 protein spots were significantly down-regulated and up-regulated in the experimental group, respectively (Additional file 3: Table S3).

### Functional classification of identified DEPs and KEGG analysis

**Fig. 4:**
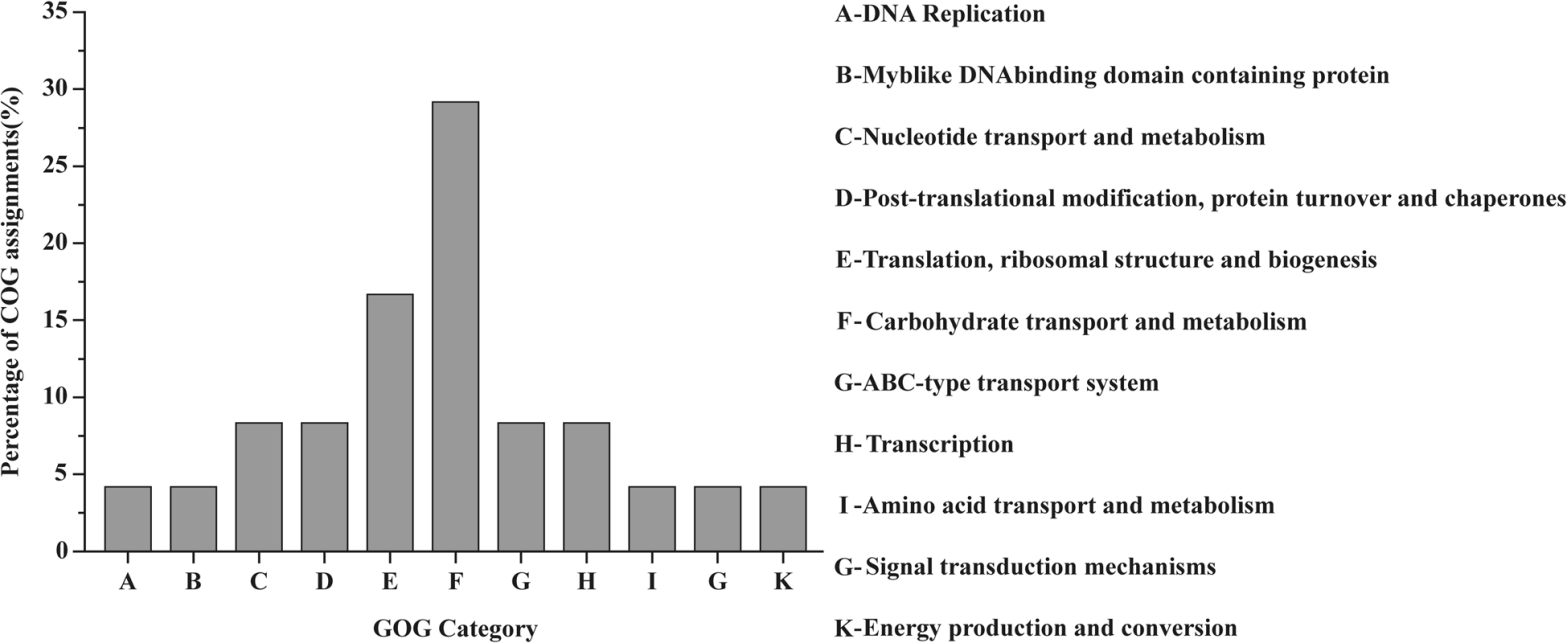
Percentage distribution of proteins by cluster of orthologous groups

COG categories were performed to divide the experimentally identified proteins into cellular roles based on the molecular functions in this study (Additional file 1: Table S2). As shown in Fig. 4, 24 DEPs were grouped into carbohydrate transport and metabolism (seven spots); translation, ribosomal structure and biogenesis (four spots); nucleotide transport and metabolism, ABC-type transport system, transcription and post-translational modification, protein turnover and chaperones (two spots each); DNA replication, myb-like DNA binding domain containing protein, amino acid transport and metabolism, signal transduction mechanisms and energy production and conversion (one spot each). Among the proteins function category, those involved in carbohydrates metabolism and energy production comprised a great deal of the identified DEPs.

As shown in Additional file 4: Figure S2, 13 DEPs were categorized into 13 KEGG pathways, in which 3 and 10 DEPs were significantly down-regulated and up-regulated, respectively. Moreover, significantly down-regulated DEPs were associated with eight KEGG pathways, including ribosome, nitrogen metabolism, quorum sensing, metabolic pathways, biosynthesis of amino acids, cysteine and methionine metabolism, glycolysis/gluconeogenesis and carbon metabolism. However, representative pathways associated with the significantly up-regulated DEPs were investigated, including ribosome, quorum sensing, metabolic pathways and biosynthesis of amino acids.

### Transcriptional expression analysis by qRT-PCR

**Fig. 5:**
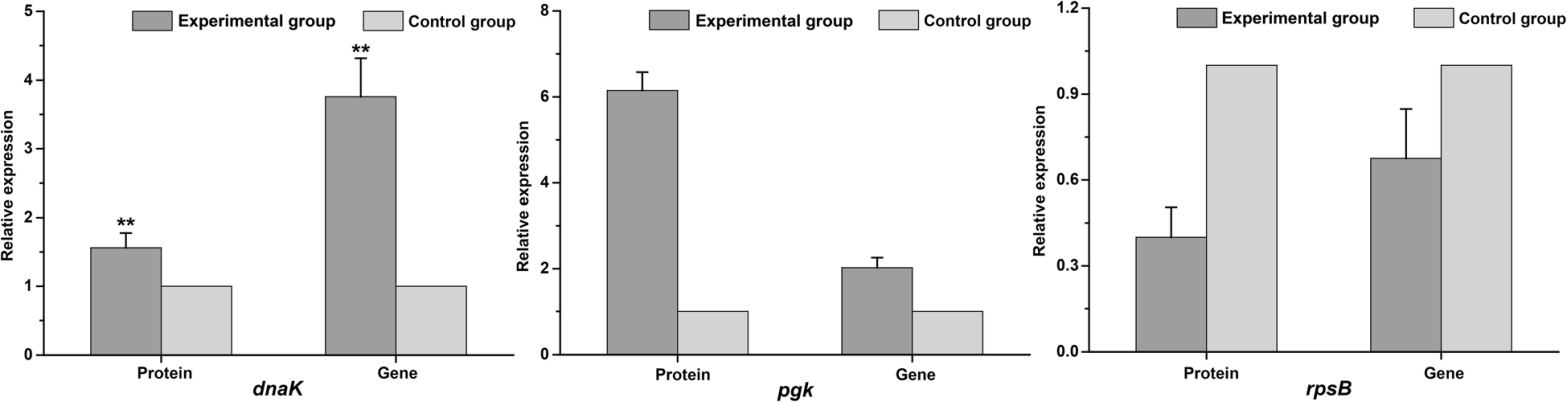
Trend graph of corresponding gene transcriptional expression was shown by qRT-PCR analysis in *Lactobacillus plantarum* cells. * indicates significant differences between the experimental and control groups. *p < 0.05 and **p < 0.01 compared with the control group by *T-*test

To provide further information of the correspondence between abundance of proteins and the transcript level of mRNA, transcriptional analysis of three proteins was performed by qRT-PCR, including molecular chaperone (DnaK), phosphoglycerate kinase (Pgk) and 30S ribosomal subunit protein S2 (RpsB) (Fig. 5).

Generally, the expression levels of DnaK, Pgk and RpsB from 2-DE data were consistent with mRNA levels (Fig. 5). Accordingly, the mRNA levels of DnaK, Pgk and RpsB by qRT-PCR determination displayed 3.8-fold, 2.0-fold and 0.6-fold, while these DEPs were 1.56-fold, 6.15-fold and 0.41-fold, respectively. In addition, according to COGs, DnaK (L07), Pgk (L18) and RpsB (L02) belonged to COG-D (post-translational modification, protein turnover, and chaperones), COG-E (carbohydrate transport and metabolism) and COG-F (translation, ribosomal structure and biogenesis), respectively, while COG-D, COG-E and COG-F showed the highest abundance in this study (Fig. 4).

## Discussion

Recently, grass carp fermentation by *L. plantarum* have been well studied, and it has been proven to be effective in the deodorization and aroma during fermentation [9]. Therefore, in the present study, we applied the integrated metabolic and proteomic approach for the study of the central metabolites and protein expression, and further elucidated the changes between intracellular substances and the corresponding fermentation mechanism by *L. plantarum*.

**Fig. 6:**
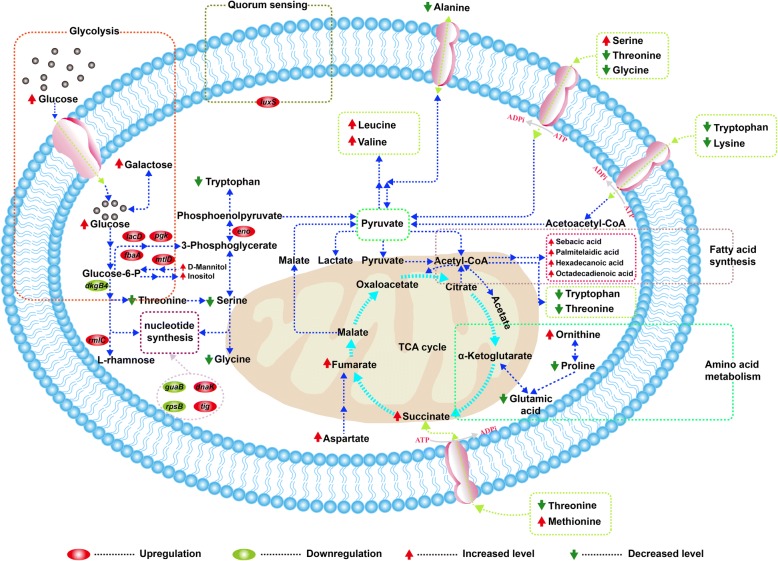
A pathway model of primary metabolic changes in *Lactobacillus plantarum*. Red arrows denote increase (*p* < 0.05) and green arrows denote decrease (*p* < 0.05)

In the current study, some pathways related to the intracellular metabolites of *L. plantarum* were shown in Fig. 6, which were mainly involved in carbohydrate metabolism, fatty acid metabolism and amino acid pathway. Among them, organic acids accounted for approximately 38 %, and most of which were related to fatty acid metabolism and TCA cycle. As shown in Fig. 6, palmitelaidic acid and oleic acid both showed a clear increasing trend compared with the control group. It was reported that they were originated from the desaturation of saturated fatty acids [24]. Also, phosphoric acid had a slight increase in the experimental group, which was involved in the regulation of signal transduction pathway as an important intermediate metabolite. Hohmann et al. reported that phosphoric acid could accumulate in cells by forming osmotic pressure to activate the high osmolarity glycerol pathway [25].

Subsequently, 20 % of amino acids were identified as shown in Additional file 1: Table S2, and they could be produced from a few intermediates during TCA cycle and glycolysis pathway. Previous studies have shown that alanine, glycine, serine and lysine can provide a sweet taste, and glutamic acid and leucine are associated with savoury and bitter taste, respectively [26]. In this study, serine and threonine showed a clear decreased trend compared with the control group (Fig. 6). It reported that serine could be converted into pyruvate through serine deaminase, while threonine could be degenerated into acetaldehyde and volatile fatty acids as a major flavour component of yogurt [27]. Additionally, the increased content of asparate and the decreased content of alanine were appeared in the experimental group. Some researches suggested that asparate could be catalyzed into some metabolites, such as alcohol and sugar compounds by three enzymes [28]. Besides, other kinds of compounds, such as alcohols, amines, esters, etc., were also appeared in the two groups. Jangeun et al. reported that these compounds could contribute to the formation of the secondary metabolites that played a key role in the flavor components [29]. Moreover, xylitol could be involved in the conversion of pentose and glucuronic acid, and d-mannitol might participate in fructose and mannose metabolism, and inositol could primarily be involved in galactose metabolism. Taken together, the significant alteration for the main metabolites might reveal its key role in a number of metabolic pathways for regulating adaptation of *L. plantarum* cells to the fermentation environments. Therefore, the decreased glutamic acid and increased aspartate, succinate and fumarate meant the disturbance in energy metabolism.

In addition, proteomics tool was used to further reveal the change of protein expression in the grass carp fermentation. In general, proteomics analysis based on COGs and KEGG pathways suggested that the fermentation by *L. plantarum* could be associated with the multiple metabolic pathways, including carbohydrate transport and metabolism, ribosome and nucleotide metabolism, chaperones, transcription and transport system, amino acid transport and metabolism, energy production and conversion as well as signal transduction mechanism. Carbohydrate metabolism involved in the generation of ATP could maintain the intracellular microenvironment homeostasis, including redox balance, ion transport, and osmoregulation [30]. In this work, proteins (FbaA (L14), LacD (L15), Pgk (L18), MtlD (L26), GpmA (L12), DkgB4 (L21), and Eno (L22)) directly or indirectly related to glycolysis and gluconeogenesis were perturbed, the first four proteins were up-regulated whereas the left down-regulated. Figure 6 summarized the proteomic and metabolomic responses involved in the pathways. Among them, FbaA, LacD, Pgk and MtlD were up-regulated to the level of 4.37-fold, 2.16-fold, 6.15-fold and 3.39-fold in the experimental group, respectively. FbaA mainly found in prokaryotes and fungi, were homodimeric enzymes and also belonged to one of the glycolytic enzymes (Fig. 6). LacD belonged to a key enzyme and can catalyze the conversion of d-tagatose 1,6-diphosphate into glyceraldehyde 3-phosphate in galactose metabolism, which subsequently participated in glycolysis. Wu et al. reported that the up-regulation of LacD could be attributed to the fact that cells produced ATP to support the extrusion of H^+^ under acid stress in *L. casei* [31]*.* Meanwhile, Pgk and DkgB4 were involved in energy and carbohydrate metabolism by glycolysis and gluconeogenesis pathway. As depicted in Fig. 6, Pgk, a monomeric two domain enzyme and the enzyme responsible for the first ATP-generating step of glycolysis, participated in energy metabolism of glycolysis, and then its main function was to generate ATP, and increased the respiration rate [32]. In addition, qPT-PCR was performed to measure the 2-fold change expression, which might be responsible for enhanced ATP synthesis that required for energy metabolism (Fig. 5). These results were approximately in agreement with the differences in carbon source, and thus in the experimental group it was reasonable to assume that sucrose catabolite could promote the regulation of the corresponding genes by *L. plantarum*.

Also, eight significantly DEPs were mainly involved in ribosome and nucleotide metabolism in *L. plantarum*, including four up-regulated and four down-regulated proteins (Additional file 3: Table S3). Ribosomal proteins such as RpsB, RplA, RpsJ and RpsQ might be related to ribosomal structure, translation, and biogenesis. Among them, RpsB and RplA proteins were found to be down-regulated to 0.40-fold and 0.44-fold, whereas RpsJ and RpsQ were up-regulated to the level of 1.52-fold and 4.49-fold during the fermentation. It has been reported that ribosomal proteins are considered as sensors of cold and heat shock, and as an essential component of translation machinery [33]. Thus, it is assumed that the results in this study can imply a relationship between acid response and multiple ribosomal protein from *L. plantarum*. Moreover, AAW28_04515 (L01, 1.58-fold) and NusA (L05, 0.32-fold) were classified as DNA replication and myb-like DNA binding domain containing protein on the basis of COGs function prediction (Additional file 3: Table S3). Zhao et al. demonstrated that DNA-binding proteins were a family of proteins induced in microorganisms by oxidative or nutritional stress [34]. Then, NusA played a crucial role in regulating gene expression in prokaryotes [35]. In addition, RmlC (L04, 3.63-fold) and GuaB (L27, 0.38-fold) were both involved in nucleotide transport and metabolism (Additional file 3: Table S3). RmlC involved in biosynthesis of dTDP-l-rhamnose is an essential component of the bacterial cell wall. GuaB is crucial for DNA and RNA synthesis, signal transduction, energy transfer, glycoprotein synthesis, as well as cellular proliferation. It was said that GuaB was a purine biosynthetic enzyme and a regulator of nucleotide synthesis, and then played a prominent role in regulating the cell growth. Besides, GuaB was down-regulated in the experimental group, which suggested that the cells of *L. plantarum* could reach saturation and stop the cellular proliferation in the stationary phase during the fermentation.

Furthermore, molecular chaperones can protect proteins in intracellular environment from some irreversible aggregation under cellular stress [36]. In the current study, DnaK (L07, 1.56-fold) and Tig (L19, 3.24-fold) proteins were both up-regulated, and both of them were stress response proteins (Additional file 3: Table S3 and Fig. 6). Then, DnaK is an enzyme involved in ATP binding, hydrolysis and ADP release. It was said that DnaK played a major role in maturation of synthesized proteins as well as protein degradation and repair, and it up-regulated in *L. casei* Zhang under acid stress condition [37]. Tig is an ATP-independent chaperone, and displays chaperone and PPIase activities in vitro [38]. In this study, the proteins of DnaK and Tig were both up-regulated in *L. plantarum*, which could be attributed to the existence of salt and accumulation of organic acids in the experimental group. Additionally, LuxS (L09) up-regulated to the level of 3.15-fold in the present study, was involved in signal transduction metabolism. Previous studies had shown that LuxS belonged to quorum sensing that regulated gene expression in response to changes in cell density, and played a central metabolic important role in cells growth and biofilm formation [39]. Also, LuxS was an autoinducer-production protein that had a metabolic function [40]. Additionally, it reported that *luxS* gene could regulate acidic stress under environmental stresses, which had been also proven in *Lactobacillus* sp*.* [41]. Therefore, compared to the control group, LuxS up-regulation could be related to the acidic and salt stress response. LCAUCD174_0314 (L17, 0.32-fold), involved in energy production and conversion, is one of aldo/keto reductase family proteins. Ehrensberger et al. reported that such proteins could catalyze reversible reduction of carbonyl-containing compounds to some corresponding alcohols in *Bacillus subtilis* [42]. In this study, aldo/keto reductase family oxidoreductase was down-regulated, and could be related to the lack of carbon source during the post-fermentation by *L. plantarum*.

In general, the alterations in the concentration of metabolites were mainly involved in glycolysis pathway, pentose-phosphate pathway, fatty acid synthesis pathway, amino acid metabolism and TCA cycle. Further, bioinformatics analysis of DEPs illustrated the enrichment of metabolism related to glycolysis/gluconeogenesis pathway, ribosome metabolism and biosynthesis of amino acids etc. Simultaneously, Fig. 6 provided an overview on the relationship between proteomic and metabolomic responses involved in the pathways. Apparently, analysis of the central carbon metabolism revealed that *L. plantarum* was able to utilize glucose via glycolysis and pentose-phosphate pathway, which was involved in four up-regulated DEPs (LacD, Pgk, FbaA and MtlD) and one down-regulated DEP (DkgB4) as well as several decreased amino acids (threonine, serine and glycine) (Fig. 6). This result suggested that *L. plantarum* could enhance the catabolism of glucose though these up-regulated proteins, and reduce the conversion of some amino acids coupled with sugar metabolism. The protein Eno participated in pentose phosphate pathway, and its up-regulation could contribute to the accumulation of pyruvate, while alanine could be also converted to pyruvate [43]. The increase of serine, aspartate and methionine can enter into the TCA cycle, through pyruvate, fumarate and succinate, respectively (Fig. 6). Also, pyruvate played a critical role in fatty acid synthesis, which was mainly associated with several kinds of polyunsaturated fatty acids (PUFAs) in *L. plantarum* as shown in Fig. 6. It reported that PUFAs such as palmitelaidic acid and oleic acid, were formed by oxygen- and NADH-dependent desaturation of octadecanoic acid and hexadecanoic acid [44]. Furthermore, the increase of these MUFAs could cause of more membrane fluidity, which suggested that it was essential to remaining *L. plantarum* cell viability by maintaining membrane fluidity [45].

This study shows that integration of metabolomics and proteomics can produce complementary data that advance the understanding regarding correlation of fermentation process. Nevertheless, there are still several limitations in the current study. The most obvious problem was that only 90 metabolites were identified in the two groups, which might be attributed to some volatile metabolites and high molecular weight metabolites being missed. Moreover, 2D-electrophoresis proteomics could not completely identify and quantify all the intracellular proteins. Thus, more advanced and sensitive high-throughput omics technologies should be used to describe the entire regulatory network.

## Conclusion

In the current study, metabolomics coupled with proteomics approach were integrated into discovering differential metabolites and proteins, and elucidating *L. plantarum* fermentation mechanism and further illuminating the molecular mechanisms of metabolites and protein changes during the grass carp fermentation. More than 80 intracellular metabolites were detected using GC-MS, while 27 significantly DEPs were determined and analyzed via proteomics, with 15 up-regulated and 12 down-regulated proteins. Based on metabolomics and proteomics analysis, we should point out that the up-regulated proteins associated with glycolysis pathway, such as LacD, FbaA, Pgk, and Eno, can contribute to enhancing TCA cycle, which is closely related to the decreased intracellular metabolite of glutamic acid and the increased accumulation of fatty acid metabolites. In addition, further studies will be required to determine the changes of some enzymatic activities by using biochemical and other approaches, and define the specific molecular pathways involved in the grass carp fermentation.

## Methods

### Materials and general methods

Grass carp of 2.0 ± 0.5 kg body weight and 60 ± 5 cm body length was purchased from a local market (Ningbo, China). Immediately after sacrificed, fresh grass carp were removed their scales, head, and gut. Then, they were rinsed in sterile deionized water, and cut into fillets approximately 40 mm thick using sterilized scissors under sterile conditions. After 15 min of UV irradiation, grass carp muscles were used for the subsequent fermentation experiment.

Pyridine (> 99.8% purity), methoxylamine hydrochloride (> 99.8% purity), BSTFA + 1% TMCS (> 99.0% purity), n-heptane (> 99.0% purity) and n-docosane (> 99.0% purity) were purchased from Sigma-Aldrich (St. Louis, MO, USA), in which pyridine and n-heptane were chromatographic grade, while other reagents were analytical grade. Then, the methoxyamine hydrochloride was dissolved in pyridine at a concentration of 15 mg/mL.

LAB was isolated from the traditional pickles in Ningbo City, and initially screened by using the skimmed milk treadmill test, followed by further rescreening and performing a series of protease activity assays experiment. Subsequently, the strain with high productivity of protease was determined by 16S rRNA sequencing and the alignment using the BLAST search program (NCBI), and 16S rRNA gene sequence of primer was shown in the supplementary material (Additional file 5). The primers used for sequencing were universal primers (27F: 5′-CAG CGG TAC CAG AGT TTG ATC CTG GCT CAG-3′; 1492R: 5′-CTC TCT GCA GTA CGG CTA CCT TGT TAC GAC TT-3′).

In the experiment of two 2-DE pH 4–7 IPG gel strips, 2-DE electrophoresis equipments and a variety of reagents were Bio-Rad company products and American Sigma company products.

### Preparation of biosamples

*L. plantarum* in 20% (*v*/v) glycerol was stored at − 80 °C, and was revitalized in de Man Rogosa and Sharpe (MRS) broth at 35 °C on a shaker at 120 rpm for 24 h before use. Then, the pH and bacterial density at OD_600nm_ were measured according to Liu et al [8]. Subsequently, *L. plantarum* for the control group was incubated in MRS broth at 35 °C for 24 h with shaking. Grass carp fillets, pickled with 50 g salt kg^− 1^ and 30 g sucrose kg^− 1^ for 4 h at room temperature under the sterile conditions, were split into three batches. Each batch was wrapped with 100 mesh gauze of four layers, and tied with sterile cotton thread. Then, they were inoculated with *L. plantarum* to a concentration of approximately 10^7–8^ cfu/mL and fermented at 35 °C for 24 h. At the end of the fermentation, the fermentation broth was immediately centrifuged at 4 °C and 1200 rpm for 10 min to remove of the pellet, and then the supernatant was centrifuged at 12,000 rpm for 10 min at 4 °C to harvest the cells. Then, the cells in each of the two groups were washed three times with sterile water and used for metabolomic and proteomic analysis.

### Extraction and derivatization of metabolites

Metabolome samples of *L. plantarum* were prepared with some modifications according to a previously described method [46]. Cells were inactivated with liquid nitrogen and fully ground, and metabolites were extracted by addition of 15 mL of 60% methanol (*w*/*v*, − 20 °C) to the cell pellet [47]. After centrifugation at 12,000 rpm for 10 min at 4 °C, supernatants were dispensed into centrifuge tubes and dried for approximately 2 h by using nitrogen to remove the excess methanol. Thereafter, the metabolites were completely frozen overnight (approximately − 40 °C), and then freeze-dried for approximately 48 h and preserved in the dryer for analyzing the metabolite levels by GC-MS.

For derivatization, a 200 μL of 15 mg/mL methoxyamine pyridine hydrochloride was added, and then mixed for 30 s prior to incubation at 37 °C for 90 min. Then, the samples were further derivatized with the addition of 200 μL BSTFA with 1% TMCS and incubated at 70 °C for 30 min, following by incubating at room temperature for 30 min [48]. After adding 100 μL of n-heptane (containing 0.10 mg/mL of n-docosane, internal standard) and vortexing, followed by centrifugation (12,000 rpm, 4 °C, 10 min), the derivatized samples were transferred to sample bottle for GC-MS analysis.

### GC-MS analysis

The GC-MS system consisted of an Agilent 7890/M780EI gas chromatograph (GC, Agilent Technologies, Palo Alto, CA, USA) coupled with a PERSEE mass spectrometer (MS, Shimadzu, Kyoto, Japan) and an Agilent AS-2912 autosampler. A sample of 1.0 mL was injected into a deactivated and fused-silica Agilent DB-5MS capillary column (30 m × 0.25 mm × 0.25 μm film, Agilent J&W Scientific, Folsom, CA) with a split ratio of 8:1. After sample preparation, the electron impact (EI) ion source temperature was set to 250 °C with a 70-eV electron beam. Then, the injector temperature was 280 °C with the detector voltage of 0.96 kV and a solvent delay of 6 min. The helium carrier gas (99.999%) was set at a flow rate 1.0 mL/min. The GC oven was held at 90 °C for 3 min, and ramped at 3 °C/min to 160 °C, then further ramped to 220 °C at 2 °C/min, where it was held for 1 min, eventually ramped at 10 °C/min to 290 °C [49]. Masses were acquired in a full scan mode over the range from 45 to 550 m/z with a 0.2 s scan velocity.

### Protein extraction and quantification

The lysis buffer (8 M Urea, 2 M Thiourea, 4% (*w*/*v*) CHAPS, 1% DTT, 0.25% (w/v) Tris) was added (volume ratio of lysis buffer to the mass of cells = 5:1) into an equal amount of cells in the two groups. The cells were resuspended and lysed in lysis buffer for 30 min at 4 °C, and subsequently disrupted by sonication at 200 W for 20 min (Ningbo Scientz Biotechnology, China). The protein preparation was performed according to previously described [50]. Protein pellets were dissolved with rehydrating buffer solution (8 M Urea, 4% (*w*/*v*) CHAPS, 2 M thiourea, 10 mg/mL DTT), and collected by centrifuging at 12,000 rpm for 10 min at 4 °C. Eventually, proteins were purified using the 2-D Clean-Up kit (GE healthcare, USA), and the protein concentration was measured by using the Bradford assay [51].

### 2-DE analysis

After determining the protein concentration from the control and experimental groups, the proteins were diluted with IEF buffer (8 M Urea, 4% (*w*/*v*) CHAPS, 2 M thiourea, 65 mM DTT, 0.2% (*v*/v) Bio-lyte (3/10, Bio-Rad, USA)). After centrifugation at 12,000 rpm and 4 °C for 10 min, isoelectric focusing (IEF) was performed on PROTEAN IEF cell (Bio-Rad, USA) by rehydrating Ready Strip IPG Strips (pH 4–7, 7 cm) with 150 μL protein solution (300 μg) for 14 h with a maximum current setting of 50 mA/strip at 20 °C. After rehydrating, the voltage program of IEF was followed by a linear ramp to 250 V over 1 h, linear ramp to 500 V over 2 h, another linear ramp to 1000 V over 1 h, then linear ramp to 4000 V over 4 h and a constant 4000 V hold for 5.6 h (thus yielding a total of 20,000 V/h). Afterwards, the immobilized pH gradient (IPG) strips were equilibrated by 2.5 mL of equilibration buffer solution I (6 M Urea, pH 8.8, 75 mM Tris-HCl, 20% Glycerol, 2% (w/v) SDS, 2% (w/v) DTT, 0.002% (w/v) bromophenol blue) for 15 min, followed by a second 15-min equilibration step in 2.5 mL of equilibration buffer solution II (6 M Urea, pH 8.8, 75 mM Tris-HCl, 20% Glycerol, 2% (w/v) SDS, 0.002% (w/v) bromophenol blue, 2.5% (w/v) iodoacetamide). After equilibrating, the IPG strips were then loaded onto 12% SDS-PAGE gels and sealed with 0.5% agarose. Then, 2D SDS-PAGE was performed by using a PowerPac basic electrophoresis system (Bio-Rad, California, USA) at 100 constant volts and 15 °C. Hereafter, gels were rinsed twice by using the double distilled water (ddH_2_O), and the protein spots were visualized by using Coomassie Brilliant blue R-250 staining. Finally, these gels were rinsed several times with ddH_2_O on a shaker.

### Image analysis and protein identification

Three parallel gels were consistent duplicates for each the control and experimental groups, and scanned with an Image Scanner (Calibrated Densitometer GS800, Bio-Rad, USA) in a transmission mode. Subsequently, PDQuest software (version 8.0.1, Bio-Rad, California, USA) was used for analyzing 2D protein profiles, and these DEPs were extracted for following identification by MALDI-TOF/TOF MS (*p* < 0.05). The pretreatment of selected protein spots and MS analysis was performed as described previously [52]. Briefly, α-cyano-4-hydroxycinnamic acid as matrix was used to ionize peptides. MS analysis of peptide solutions from trypsin digested proteins was performed on a Bruker UltraReflex™ III MALDI-TOF/TOF mass spectrometer (Bruker Daltonics, Karlsruhe, Germany) [22]. A maximum accelerating potential of 20 kV was used in this instrument with UV wavelength of 355 nm and repetition rate of 200 Hz. It was operated in reflector mode and scanning quality range was 700–3200 Da, while the best quality resolution was 1500 Da. The pancreatin self-cutting peak was internal standard to correct the mass spectrometer, and the mass spectra of samples were obtained by default mode. Peptide mass fingerprints were processed by using a software flex analysis (Bruker Daltonics, Germany). The MS data were interpreted by BioTools 3.0 (Bruker Daltonics, Germany) combined with the Mascot search engine against Uniprot database for known proteins from firmicutes. A fixed modification of cysteine was implemented by allowing for one missed cleavage site and assuming carbamidomethyl, and with the oxidized methionine as a variable modification. Then, peptide mass tolerance and fragment mass tolerance were set to 50 ppm and ± 0.6 Da, respectively [22].

### Multivariate analysis

All GC-MS data, including peak intensities, retention characteristics and the integrated mass spectra of each cell metabolites sample, were used for the component analysis [53]. Based on the similarity of their retention time, the commensal metabolites of each sample were aligned. Then, in accordance with each GC-MS TICs, the areas of corresponding chromatographic peaks were determined. The identification of peaks-of-interest was performed with their mass spectra by matching with NIST08 MS spectral library of the WILEY workstation (similarity ratio > 80%). The concentrations of metabolites were represented as the relative areas (divided by the area of reference compounds). The preprocessed GC-MS data were subsequently imported into Simca-P software (Demo version 11.0, Umetrics, Umea, Sweden), and subjected to the principal component analysis (PCA) and loadings analysis. Finally, a heatmap of these metabolites was performed using HemI 1.0 software [54].

UniProt protein sequence database was used to determine the theoretical pI values and molecular weights (MWs) of the identified proteins [55]. Also, the assembled transcripts were searched using BLASTx (NCBI database) with a cut-off E-value of 1E-5 [50]. Then, the identified proteins were distributed over clusters of orthologous groups (COGs), and grouped into the cellular roles according to COGs. Additionally, the proteins were further defined according to Kyoto Encyclopedia of Genes and Genomes (KEGG, http://www.genome.jp/kegg/).

All the experiments were performed in triplicate. Then, *T*-test was used to analyze the significance between the control and experimental groups. A *p*-value of < 0.05 in all replicate experiments represented statistical significance.

### Quantitative real-time polymerase chain reaction (qRT-PCR)

Total RNA was extracted and purified from frozen cell pellets using the RNeasy mini RNA extraction kit (Trizol, Invitrogen, USA) in accordance with the manufacturer’s instructions, and then RNA yields were determined by using a NanoDrop 2000 Spectrophotometer (Thermo Fisher Scientific Inc. South Logan, USA). Total RNA based on the manufacturer’s protocol was reverse-transcribed into the first strand cDNA synthesis by using oligo (dT) primers and the M-MLV first strand cDNA synthesis kit (Invitrogen, USA). A Primer3plus software was used to design qRT-PCR analysis primers based on the genome sequence of *L. plantarum* (Additional file 6: Table S1).

qRT-PCR was performed by a Rotor-Gene 6000 real-time PCR detection system (Corbett Research, Mortlake, Victoria, Australia) along with fluorescence signal detection (SYBR® Premix Ex TaqTM II) with four biological replicates. A 20.0 μL reaction mixture was used for each PCR reaction, including 10.0 μL of SYBR® Premix Ex TaqTM II (2X), 2.0 μL of properly diluted cDNA (30 ng/μL of cDNA for all genes used for qRT-PCR except for 16S rRNA, where the concentration of cDNA was 0.3 ng/μL), each forward and reverse primer (10 μM) of 1.0 μL, and 6.0 μL of nuclease-free water [56]. The negative control in each run was set to Diethyl pyrocarbonate (DEPC)-treated water. The thermal cycling conditions contained an initial denaturation step for 95 °C for 10 s, followed by 40 cycles of 15 s denaturation at 94 °C, annealing (10 s at 54 °C) and extension (15 s at 72 °C), followed by a single fluorescence measurement [57].

A specific threshold for each gene was set to analyze the data at the approximate midpoint of exponential phase to the amplification. Each of four biological replicates on the resulting threshold cycle (Ct) values were averaged. Subsequently, the standard curves were established to evaluate the expression levels of target genes, and eventually Ct values were converted to the relative amounts of cDNA [58].

## Additional files

Additional file 1: **Table S2.** The intracellular metabolites of *Lactobacillus plantarum* identified by GC-MS that differ between the control and experimental groups. A total of 90 endogenous metabolites were identified by GC-MS in this study. (DOCX 33 kb)

Additional file 2: **Figure S1.** Multivariate statistical analysis for the metabolites between the control and experimental groups. 82 metabolites between the control and experimental groups were used to build a PCA model and loadings plot. (DOCX 387 kb)

Additional file 3: **Table S3.** Identification of differentially expressed proteins in *Lactobacillus plantarum* was employed by using MALDI-TOF-MS/MS. 11 and 16 protein spots were significantly down-regulated and up-regulated in the experimental group, respectively. (DOCX 28 kb)

Additional file 4: **Figure S2.** Classification of the identified proteins by KEGG database, and the thirteen most significant KEGG pathways in *Lactobacillus plantarum*. (DOCX 93 kb)

Additional file 5: 16S rRNA gene sequence of *Lactobacillus plantarum*. The strain with high productivity of protease was determined by 16S rRNA sequencing. (DOCX 14 kb)

Additional file 6: **Table S1.** The primer sequences for qRT-PCR. A Primer3plus software was used to design qRT-PCR analysis primers based on the genome sequence of *Lactobacillus plantarum*. (DOCX 15 kb)

## Abbreviations

2-DE: Two-dimensional electrophoresis
BSTFA: N,O-bis(trimethylsilyl)trifluoroacetamide
COGs: Clusters of orthologous groups
Ct: Threshold cycle
EI: Electron impact
GC-MS: Gas chromatography-mass spectrometry
KEGG: Kyoto Encyclopedia of Genes and Genomes
LAB: Lactic acid bacterium
MRS: Man Rogosa and Sharpe
MWs: Molecular weights
PCA: Principal component analysis
qRT-PCR: Quantitative real-time polymerase chain reaction
TICs: Total ion current chromatograms
TMCS: Trimethyl-chlorosil-ane
