# Supplementary material for: A metabolomics and proteomics study of the Lactobacillus plantarum in the grass carp fermentation
Source: BMC Microbiol. 2018 Dec 18;18:216. doi: 10.1186/s12866-018-1354-x (PMC6299570; doi:10.1186/s12866-018-1354-x)
Supplement: Supplementary file 1 — Table S2. The intracellular metabolites of Lactobacillus plantarum identified by GC-MS that differ between the control and experimental groups. A total of 90 endogenous metabolites were identified by GC-MS in this study. (DOCX 33 kb) [file 12866_2018_1354_MOESM1_ESM.docx]

**Table S2.** The intracellular metabolites of *Lactobacillus plantarum* identified by GC-MS that differ between the control and experimental group. Concentrations were presented as mean ± SD; and “-” represented the uncertain endogenous metabolites. ^a^*p* < 0.05 and ^b^*p* < 0.01 compared with the control.

| Retention  time(min) | Endogenous metabolites | Formula | Concentration (μg/μL) | | *p*-value | -lg(*p*) | Change |
| --- | --- | --- | --- | --- | --- | --- | --- |
|  |  |  | Control  group | Experimental group |  |  |  |
| 6.072 | Alanine^b^ | C_3_H_7_NO_2_ | 0.114±0.011 | 0.031±0.002 | 0.001435 | 2.843010 | ↓ |
| 6.433 | 3-Oxaoct-4-en-2-imine^b^ | C_6_H_9_NO | 0.069±0.004 | 0.031±0.003 | 2.91E-04 | 3.536093 | ↓ |
| 6.618 | β-Amino isobutyric acid^b^ | C_4_H_9_NO_2_ | 0.131±0.008 | 0.034±0.002 | 3.10E-04 | 3.508384 | ↓ |
| 6.906 | Diethylcarbamate | C_5_H_12_N_2_O_2_ | 0.047±0.006 | 0.033±0.003 | 0.551310 | -0.2583 | ↓ |
| 7.133 | Ethanedioic acid^b^ | C_2_H_2_O_4_ | 0.038±0.002 | 0.049±0.003 | 0.001020 | 2.991400 | ↑ |
| 7.648 | Leucine^b^ | C_6_H_13_NO_2_ | 0.005±0.001 | 0.007±0.001 | 0.001398 | 2.854453 | ↑ |
| 7.823 | Benzoic acid | C_7_H_6_O_2_ | 0.025±0.002 | 0.015±0.001 | 0.285094 | -0.54501 | ↓ |
| 8.256 | 10-Undecenoic acid^b^ | C_11_H_20_O_2_ | 0.059 | 0.055±0.002 | 0.003246 | 2.488713 | ↓ |
| 9.492 | Valine^b^ | C_5_H_11_NO_2_ | 0 | 0.005±0.001 | 4.95E-05 | 4.305794 | ↑ |
| 11.006 | Serine^b^ | C_3_H_7_NO_3_ | 0.008±0.001 | 0.137±0.003 | 3.24E-04 | 3.489527 | ↑ |
| 12.386 | Ornithine^b^ | C_5_H_12_N_2_O_2_ | 0.066 | 0.169±0.001 | 1.37E-05 | 4.864701 | ↑ |
| 12.716 | Glycine^b^ | C_2_H_5_NO_2_ | 0.290±0.010 | 0.072±0.002 | 0.001316 | 2.880709 | ↓ |
| 13.314 | Mercaptoacetic acid^b^ | C_2_H_4_O_2_S | 0.010±0.002 | 0 | 5.52E-04 | 3.257959 | ↓ |
| 13.746 | Tartaric acid^a^ | C_4_H_6_O_6_ | 0.060±0.002 | 0.031±0.001 | 0.024652 | -1.60814 | ↓ |
| 14.014 | 4,6-Dihydroxypyrimidine^b^ | C_4_H_4_N_2_O_2_ | 0.013±0.001 | 0 | 6.28E-05 | 4.201782 | ↓ |
| 14.848 | Homoserine^b^ | C_4_H_9_NO_3_ | 0 | 0.005±0.001 | 4.31E-04 | 3.365345 | ↑ |
| 14.931 | Uncertain | - | 0.077±0.006 | 0 | 0.208712 | 0.680451 | ↓ |
| 15.899 | Threonine^b^ | C_4_H_9_NO_3_ | 0.294±0.018 | 0 | 0.001755 | 2.755779 | ↓ |
| 16.826 | Methionine^b^ | C_5_H_11_NO_2_S | 0 | 0.009±0.001 | 1.16E-04 | 3.934523 | ↑ |
| 17.464 | Aspartate^b^ | C_4_H_7_NO_4_ | 0.066±0.005 | 0.313±0.026 | 0.002956 | 2.529229 | ↑ |
| 18.392 | 5-Hydroxytryptophan^b^ | C_11_H_12_N_2_O_3_ | 0.008±0.001 | 0 | 4.66E-05 | 4.331597 | ↓ |
| 19.123 | Aminomalonic acid | C_3_H_5_NO_4_ | 0.014±0.002 | 0.012 | 0.120844 | -0.917780 | ↓ |
| 19.308 | Uncertain^a^ | - | 0.020±0.001 | 0.034±0.003 | 0.018393 | 1.735348 | ↑ |
| 19.545 | Niacinamide^b^ | C_6_H_6_N_2_O | 0.040±0.003 | 0.011±0.001 | 0.004439 | 2.352727 | ↓ |
| 19.967 | Succinate^b^ | C_4_H_6_O_4_ | 0 | 0.006±0.001 | 5.43E-05 | 4.264982 | ↑ |
| 20.462 | 2-Pyrrolidone-5-  carboxylic acid | C_13_H_23_NO_3_ | 0.013±0.001 | 0.009±0.001 | 0.759403 | -0.11953 | ↓ |
| 20.700 | D-Threitol^b^ | C_3_H_9_O_4_ | 0.018±0.001 | 0 | 6.05E-05 | 4.218472 | ↓ |
| 21.029 | Proline^b^ | C_5_H_9_NO_2_ | 0.444±0.025 | 0.192±0.022 | 0.003238 | 2.489770 | ↓ |
| 21.193 | Asparagine^b^ | C_4_H_8_N_2_O_3_ | 0.528±0.029 | 0.206±0.031 | 0.004811 | 2.317741 | ↓ |
| 21.729 | 2-Piperidinecarboxylic acid^b^ | C_6_H_11_NO_2_ | 0.140±0.010 | 0.354±0.075 | 0.005904 | 2.228864 | ↑ |
| 21.966 | Fumarate^b^ | C_4_H_4_O_4_ | 0 | 0.029±0.001 | 6.26E-04 | 3.203758 | ↑ |
| 23.357 | 2-Amino-2-propane-1,3-diol^b^ | C_2_H_7_NO_3_ | 0.011±0.001 | 0.016±0.001 | 3.55E-05 | 4.450364 | ↑ |
| 24.119 | 2-Propenoic acid^a^ | C_3_H_4_O_2_ | 0.019±0.002 | 0.008±0.002 | 0.021407 | -1.66945 | ↓ |
| 25.026 | Glutamic acid^a^ | C_5_H_9_NO_4_ | 1.307±0.208 | 0.556±0.210 | 0.027854 | -1.55512 | ↓ |
| 25.664 | β-D-glucopyranoside^b^ | C_6_H_11_O_6_ | 0 | 0.009±0.001 | 1.76E-05 | 4.754804 | ↑ |
| 25.839 | Gluconic acid^b^ | C_6_H_12_O_7_ | 0.028±0.001 | 0.009±0.001 | 0.002983 | 2.525400 | ↓ |
| 27.921 | Uncertain^b^ | - | 0.023±0.001 | 0 | 9.33E-05 | 4.029978 | ↓ |
| 28.477 | Xylitol^b^ | C_5_H_12_O_5_ | 0.008±0.001 | 0.016±0.002 | 0.009814 | 2.008144 | ↑ |
| 29.363 | 1-Aminocyclopropanecar-boxylic acid^b^ | C_4_H_7_NO_2_ | 0.011±0.001 | 0 | 1.15E-04 | 3.937889 | ↓ |
| 30.301 | Phosphoric acid^a^ | H_3_PO_4_ | 0.132±0.005 | 0.139±0.005 | 0.035821 | -1.44587 | ↑ |
| 30.858 | Ribonic acid^b^ | C_5_H_10_O_5_ | 0.009±0.001 | 0.027±0.002 | 0.002158 | 2.665946 | ↑ |
| 31.506 | Mesaconic acid^b^ | C_5_H_6_O_4_ | 0.009±0.001 | 0 | 6.64E-05 | 4.177710 | ↓ |
| 31.692 | Azelaic acid^b^ | C_9_H_16_O_4_ | 0.022 | 0.013±0.001 | 0.009406 | 2.026617 | ↓ |
| 32.125 | Propanoic acid | C_3_H_6_O_2_ | 0.250±0.050 | 0.332±0.043 | 0.170487 | -0.76831 | ↑ |
| 32.485 | 1,2,3-Propanetricarboxylic acid^b^ | C_6_H_8_O_6_ | 0 | 0.005±0.001 | 1.82E-05 | 4.741051 | ↑ |
| 32.866 | Uncertain^b^ | - | 0.054±0.006 | 0.011±0.001 | 0.003038 | 2.517385 | ↑ |
| 33.124 | Uncertain^b^ | - | 0 | 0.014±0.001 | 1.32E-04 | 3.878960 | ↑ |
| 33.278 | 2-Deoxy-galactopyranose^b^ | C_6_H_12_O_5_ | 0.028±0.001 | 0 | 6.05E-05 | 4.218553 | ↓ |
| 33.587 | α-Acetyl-Lysine^a^ | C_8_H_16_N_2_O_3_ | 0.043±0.001 | 0.067±0.011 | 0.013197 | -1.87952 | ↑ |
| 33.742 | Tetradecanoic acid | C_14_H_28_O_2_ | 0.009±0.001 | 0.005±0.001 | 0.188408 | -0.7249 | ↓ |
| 33.969 | D-Pinitol | C_7_H_14_O_6_ | 0.011±0.002 | 0.013±0.003 | 0.052460 | -1.28017 | ↑ |
| 34.195 | 2-Butenedioic acid^b^ | C_4_H_6_O_2_ | 0 | 0.011±0.001 | 3.83E-05 | 4.416445 | ↑ |
| 35.039 | D-Glucose | C_6_H_12_O_6_ | 0.013±0.001 | 0.014±0.001 | 0.705443 | -0.151514 | ↑ |
| 35.287 | D-Allose^a^ | C_6_H_12_O_6_ | 0.012 | 0.006±0.001 | 0.027245 | -1.56472 | ↓ |
| 35.432 | Talose^b^ | C_6_H_12_O_6_ | 0 | 0.007 | 4.57E-07 | 6.340036 | ↓ |
| 36.554 | Lysine^b^ | C_6_H_14_N_2_O_2_ | 0.289±0.040 | 0.026±0.002 | 0.004457 | 2.350922 | ↓ |
| 36.657 | D-Mannitol^b^ | C_6_H_14_O_6_ | 0 | 0.021±0.001 | 5.81E-05 | 4.235541 | ↑ |
| 36.936 | Galactose^a^ | C_6_H_12_O_6_ | 0.006±0.001 | 0.012±0.002 | 0.014103 | -1.85068 | ↑ |
| 37.636 | Inositol^b^ | C_6_H_12_O_6_ | 0.045±0.003 | 0.014±0.001 | 0.005842 | 2.233411 | ↓ |
| 39.243 | D-Mannose | C_6_H_12_O_6_ | 0.011±0.003 | 0.007 | 0.087513 | -1.057931 | ↓ |
| 39.676 | α-D-Glucopyranosiduronic acid^b^ | C_6_H_10_O_7_ | 0.026±0.001 | 0.001 | 3.00E-04 | 3.523240 | ↓ |
| 40.788 | 2(1H)-Pyrimidinone^b^ | C_4_H_4_N_2_O | 0.023±0.001 | 0.011±0.001 | 4.19E-04 | 3.377398 | ↓ |
| 40.953 | Palmitelaidic acid^b^ | C_16_H_30_O_2_ | 0.014±0.001 | 0.016±0.001 | 1.19E-04 | 3.924187 | ↑ |
| 41.231 | 9-Hexadecenoic acid^b^ | C_16_H_30_O_2_ | 0.014±0.001 | 0.015±0.001 | 1.58E-04 | 3.802635 | ↑ |
| 41.365 | Uncertain^b^ | - | 0.018±0.001 | 0 | 2.08E-05 | 4.681316 | ↓ |
| 42.004 | Hexadecanoic acid^a^ | C_16_H_32_O_2_ | 0.269±0.038 | 0.245±0.050 | 0.022079 | -1.65601 | ↓ |
| 43.157 | Uncertain^b^ | - | 0 | 0.005 | 1.69E-06 | 5.770909 | ↑ |
| 43.878 | D-Glucopyranose^b^ | C_6_H_12_O_6_ | 0.035±0.001 | 0.031±0.001 | 5.24E-05 | 4.281039 | ↓ |
| 44.115 | N-Acetyl-D-Glucosamine^b^ | C_6_H_13_O_5_N | 0.058±0.002 | 0.020±0.001 | 3.97E-05 | 4.401085 | ↓ |
| 44.898 | Gulose^b^ | C_6_H_12_O_6_ | 0.006 | 0.007±0.001 | 0.001282 | 2.892137 | ↑ |
| 45.424 | α-D-Glucopyranoside^b^ | C_6_H_11_O_6_ | 0.009 | 0.007 | 0.001315 | 2.880896 | ↓ |
| 46.187 | D-Ribofuranose^b^ | C_5_H_10_O_5_ | 0.012 | 0 | 4.37E-06 | 5.359991 | ↓ |
| 46.505 | α-D-Glucofuranose^b^ | C_12_H_20_O_6_ | 0.014±0.001 | 0.015±0.001 | 1.31E-04 | 3.882161 | ↑ |
| 47.196 | 1-amino-Cyclopentanecar  boxylic acid^b^ | C_6_H_11_NO_2_ | 0.010 | 0.021±0.001 | 9.21E-05 | 4.035874 | ↑ |
| 47.947 | Benzaldehyde | C_7_H_6_O_2_ | 0.025±0.004 | 0.032±0.001 | 0.196288 | -0.7071 | ↑ |
| 48.596 | 17-Octadecynoic acid^b^ | C_18_H_32_O_2_ | 0 | 0.033±0.001 | 3.60E-04 | 3.443497 | ↑ |
| 48.905 | Oleic acid^b^ | C_18_H_34_O_2_ | 0.033±0.001 | 0.081±0.006 | 0.002440 | 2.612665 | ↑ |
| 49.194 | 9-Octadecenoic acid^b^ | C_18_H_34_O_2_ | 0.104±0.010 | 0.125±0.012 | 0.002851 | 2.543636 | ↑ |
| 50.152 | Octadecanoic acid^b^ | C_18_H_36_O_2_ | 0.170±0.025 | 0.208±0.032 | 0.004010 | 2.396823 | ↑ |
| 50.731 | 9,12-Octadecadienoic acid^b^ | C_18_H_32_O_2_ | 0.012±0.001 | 0.046±0.010 | 0.004620 | 2.335372 | ↑ |
| 51.151 | 6,9-Octadecadienoic acid^b^ | C_18_H_32_O_2_ | 0.012±0.001 | 0.021±0.002 | 1.69E-04 | 3.771169 | ↑ |
| 52.367 | Uncertain^b^ | - | 0.005±0.001 | 0 | 2.11E-05 | 4.674916 | ↓ |
| 52.562 | 11,14-Eicosadienoic acid^b^ | C_20_H_36_O_2_ | 0.038±0.001 | 0.035±0.001 | 2.53E-04 | 3.597067 | ↓ |
| 53.213 | 10-Nonadecenoic acid^b^ | C_19_H_36_O_2_ | 0 | 0.009±0.001 | 2.77E-05 | 4.556959 | ↑ |
| 53.973 | D-Glycero-D-gulo-  Heptose^b^ | C_7_H_14_O_7_ | 0.044±0.002 | 0.042±0.002 | 3.39E-04 | 3.469489 | ↓ |
| 54.664 | α-D-Galactopyranose^b^ | C_6_H_12_O_6_ | 0 | 0.005±0.001 | 2.03E-05 | 4.692523 | ↑ |
| 56.693 | 1-amino-9,10-  Anthracenedione^b^ | C_14_H_9_NO_2_ | 0.010±0.001 | 0.003 | 0.008754 | 2.057786 | ↓ |
| 57.404 | Sebacic acid^b^ | C_10_H_18_O_4_ | 0.103±0.017 | 0.187±0.037 | 0.007201 | 2.142590 | ↑ |
| 58.527 | 2,5-Dihydroxyaceto-  Phenone^b^ | C_8_H_8_O_3_ | 0 | 0.007±0.001 | 5.69E-05 | 4.244997 | ↑ |
| 58.671 | 3-Indoleacrylic acid^b^ | C_11_H_9_NO_2_ | 0.023±0.001 | 0.019±0.002 | 0.004571 | 2.339919 | ↓ |
