# Supplementary material for: A metabolomics and proteomics study of the Lactobacillus plantarum in the grass carp fermentation
Source: BMC Microbiol. 2018 Dec 18;18:216. doi: 10.1186/s12866-018-1354-x (PMC6299570; doi:10.1186/s12866-018-1354-x)
Supplement: Supplementary file 2 — Figure S1. Multivariate statistical analysis for the metabolites between the control and experimental groups. 82 metabolites between the control and experimental groups were used to build a PCA model and loadings plot. (DOCX 387 kb) [file 12866_2018_1354_MOESM2_ESM.docx]

**
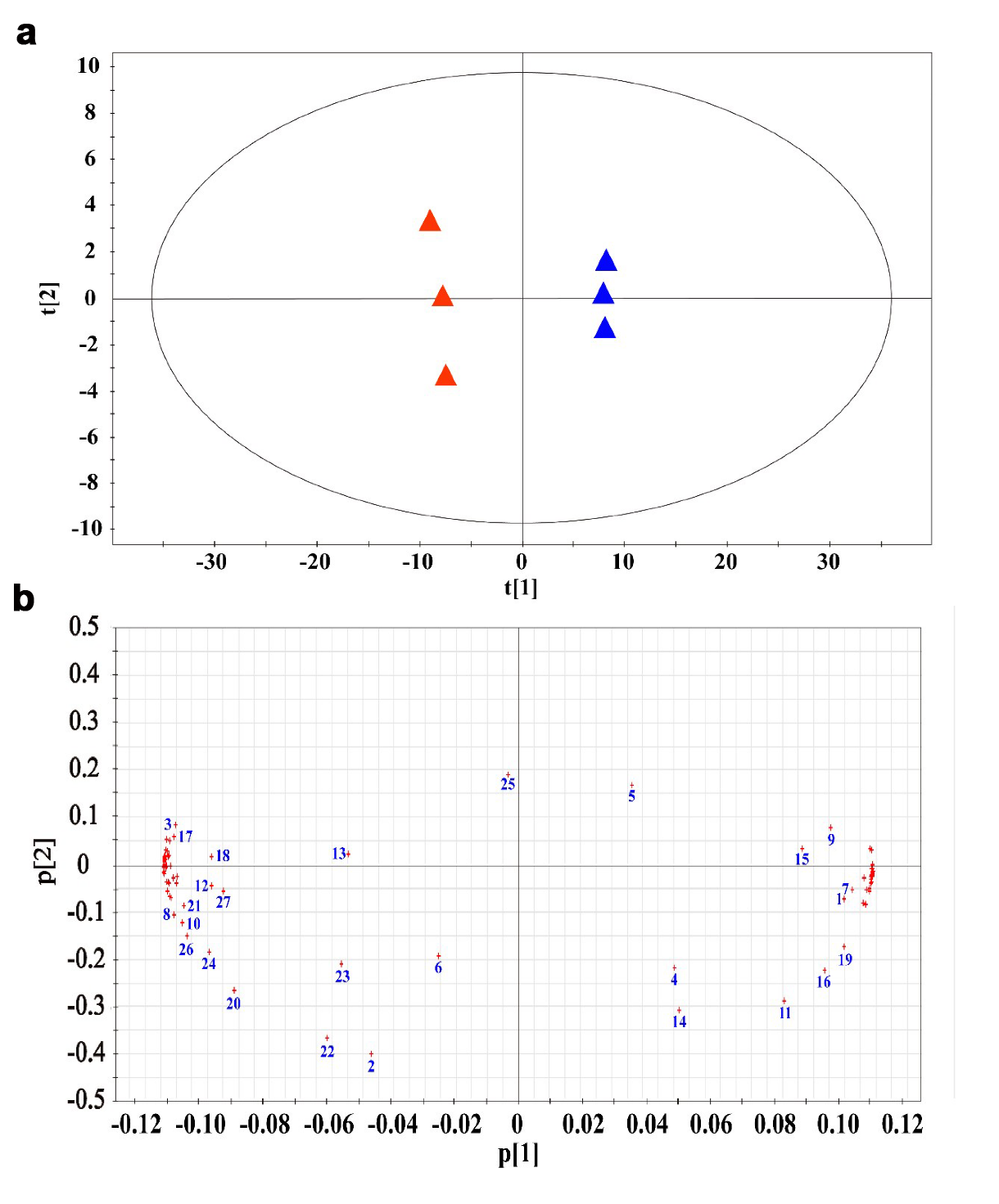
**

**Figure S1.** Multivariate statistical analysis for the metabolites between control and experimental group. **(a)** Scores plot of principal components analysis (PCA) of the control group (blue triangle) and experimental group (red triangle). The t[1] and t[2] values represent the scores of each metabolite sample in the principal components 1 and 2, respectively. **(b)** The variables’ loadings plot of the control group and experimental group from the PCA. The p[1] and p[2] values represent the contributing weights of each metabolite to the principal components 1 and 2 of the PCA model, respectively. *1* 3-Oxaoct-4-en-2-imine, *2* Diethylcarbamate, *3* Leucine, *4* Benzoic acid, *5* Aminomalonic acid, *6* 2-Pyrrolidone-5-carboxylic acid, *7* 2-Propenoic acid, *8* Phosphoric acid, *9* Azelaic acid, *10* Propanoic acid, *11* Tetradecanoic acid, *12* D-Pinitol, *13* D-Glucose, *14* D-Allose, *15* D-Mannose, *16* 2(1H)-Pyrimidinone, *17* Palmitelaidic acid, *18* D-Glucopyranose, *19* D-Glucosamine, *20* Gulose, *21* α-D-Glucopyranoside, *22* Benzaldehyde, *23* 9,12-Octadecadienoic acid, *24* 11,14-Eicosadienoic acid, *25* 10-Nonadecenoic acid, *26* D-Glycero-D-gulo-Heptose, *27* 3-Indoleacrylic acid.
