# Supplementary material for: A metabolomics and proteomics study of the Lactobacillus plantarum in the grass carp fermentation
Source: BMC Microbiol. 2018 Dec 18;18:216. doi: 10.1186/s12866-018-1354-x (PMC6299570; doi:10.1186/s12866-018-1354-x)
Supplement: Supplementary file 3 — Table S3. Identification of differentially expressed proteins in Lactobacillus plantarum was employed by using MALDI-TOF-MS/MS. 11 and 16 protein spots were significantly down-regulated and up-regulated in the experimental group, respectively. (DOCX 28 kb) [file 12866_2018_1354_MOESM3_ESM.docx]

**Table S3.** Identification of differentially expressed proteins in *Lactobacillus plantarum* was employed by using MALDI-TOF-MS/MS. Fold change was calculated using PDQuest compared to the control group. COG represented the clusters of orthologous groups.

| Spot  no. | Accession no. | Protein name (Gene) | Mascrot score | Sequence  coverage | MW/pI | Expect | Fold change | COG |
| --- | --- | --- | --- | --- | --- | --- | --- | --- |
| L01 | gi:823694945 | DNA-binding protein (AAW28_04515) | 94 | 49% | 9520/9.85 | 1.5×10^-5^ | 1.58 | DNA Replication |
| L05 | gi:406356677 | Transcription termination/antitermination protein (nusA) | 65 | 24% | 44689/4.40 | 0.012 | 0.32 | Myblike DNA binding domain containing protein |
| L04 | gi:544589596 | dTDP-4-dehydrorhamnose 3,5-epimerase (rmlC) | 405 | 49% | 21525/5.20 | 1.2×10^-36^ | 3.63 | Nucleotide transport and metabolism |
| L27 | gi:342240345 | Inosine-5'-monophosphate dehydrogenase (guaB) | 144 | 13% | 40038/5.38 | 1.4×10^-10^ | 0.38 |  |
| L07 | gi:489723390 | Chaperone protein (dnaK) | 149 | 15% | 67523/4.80 | 4.8×10^-11^ | 1.56 | Post-translational modification, protein turnover and chaperones |
| L19 | gi:823493115 | Trigger factor (tig) | 145 | 39% | 49875/4.56 | 1.2×10^-10^ | 3.24 |  |
| L02 | gi:489736644 | 30S ribosomal protein S2 (rpsB) | 151 | 16% | 30207/5.23 | 2.9×10^-11^ | 0.40 | Translation, ribosomal structure and biogenesis |
| L03 | gi:410522709 | 50S ribosomal protein L1 (rplA) | 114 | 35% | 21621/9.14 | 1.5×10^-7^ | 0.44 |  |
| L10 | gi:573005864 | 30S ribosomal protein S10 (rpsJ) | 37 | 14% | 11728/9.87 | 0.0098 | 1.52 |  |
| L25 | gi:190711126 | 30S ribosomal protein S17 (rpsQ) | 70 | 21% | 8005/9.79 | 0.0042 | 4.49 |  |
| L12 | gi:823694947 | 2,3-bisphosphoglycerate-dependent phosphoglycerate mutase (gpmA) | 130 | 22% | 25922/5.55 | 3.8×10^-9^ | 0.66 | Carbohydrate transport and metabolism |
| L14 | gi:418001045 | Fructose-bisphosphate aldolase class II (fbaA) | 123 | 23% | 31640/5.08 | 1.9×10^-8^ | 4.37 |  |
| L15 | gi:966109529 | Tagatose 1,6-diphosphate aldolase (lacD) | 202 | 28% | 36304/5.10 | 2.4×10^-16^ | 2.16 |  |
| L18 | gi:406356677 | Phosphoglycerate kinase (pgk) | 392 | 42% | 42211/5.64 | 2.4×10^-35^ | 6.15 |  |
| L21 | gi:448273061 | 2,5-diketo-D-gluconate reductase  (dkgB4) | 87 | 11% | 33413/5.50 | 7.7×10^-5^ | 0.40 |  |
| L22 | gi:823493115 | Enolase (eno) | 230 | 26% | 47039/4.70 | 3.8×10^-19^ | 0.21 |  |
| L26 | gi:716056622 | Mannitol-1-phosphate 5-dehydrogenase (mtlD) | 405 | 46% | 42615/5.27 | 1.2×10^-36^ | 3.39 |  |
| L11 | gi:380031102 | Translation elongation factor P (efp) | 66 | 10% | 20353/5.03 | 0.0099 | 1.89 | ABC-type transport system |
| L24 | gi:823694950 | Sugar ABC transporter substrate-binding protein (AAW28_07075) | 259 | 48% | 14711/5.25 | 4.8×10^-22^ | 4.23 |  |
| L20 | gi:406356677 | Cold shock protein A (cspA) | 123 | 39% | 7198/4.60 | 1.9×10^-8^ | 1.88 | Transcriptional regulators |
| L23 | gi:823694947 | XRE family transcriptional regulator (AAW28_06095) | 117 | 32% | 14163/5.49 | 7.6×10^-8^ | 0.43 |  |
| L08 | gi:823694963 | Cysteine synthase (AAW28_13085) | 216 | 40% | 32571/5.35 | 9.6×10^-18^ | 0.26 | Amino acid transport and metabolism |
| L09 | gi:932734168 | S-ribosylhomocysteine lyase (luxS) | 78 | 18% | 16977/5.82 | 0.00052 | 3.15 | Signal transduction metabolism |
| L17 | gi:770680298 | Aldo/keto reductase family oxidoreductase (LCAUCD174_0314) | 156 | 26% | 31602/5.99 | 9.6×10^-12^ | 0.32 | Energy production and conversion |
| L06 | gi:864438563 | Uncharacterized protein (LBP_cg1675) | 60 | 31% | 32683/5.20 | 0.034 | 0.38 | Unknown function |
| L13 | gi:823694950 | Uncharacterized protein (AAW28_08540) | 84 | 17% | 14576/4.87 | 0.00015 | 3.58 |  |
| L16 | gi:823694945 | Uncharacterized protein (AAW28_05315) | 84 | 38% | 14217/5.01 | 0.00014 | 3.32 |  |
