# Supplementary material for: A metabolomics and proteomics study of the Lactobacillus plantarum in the grass carp fermentation
Source: BMC Microbiol. 2018 Dec 18;18:216. doi: 10.1186/s12866-018-1354-x (PMC6299570; doi:10.1186/s12866-018-1354-x)
Supplement: Supplementary file 4 — Figure S2. Classification of the identified proteins by KEGG database, and the thirteen most significant KEGG pathways in Lactobacillus plantarum. (DOCX 93 kb) [file 12866_2018_1354_MOESM4_ESM.docx]

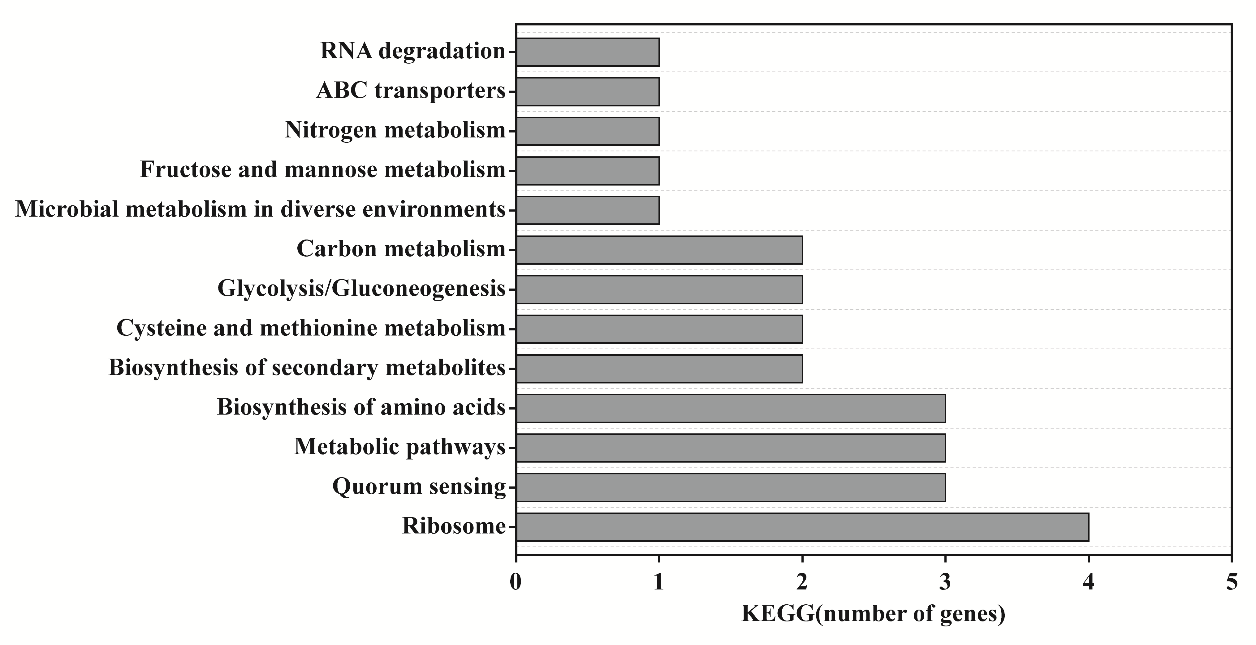


**Figure S2.** Classification of the identified proteins by KEGG database, and the thirteen most significant KEGG pathways in *Lactobacillus plantarum*.
