# Supplementary material for: A metabolomics and proteomics study of the Lactobacillus plantarum in the grass carp fermentation
Source: BMC Microbiol. 2018 Dec 18;18:216. doi: 10.1186/s12866-018-1354-x (PMC6299570; doi:10.1186/s12866-018-1354-x)
Supplement: Supplementary file 5 — 16S rRNA gene sequence of Lactobacillus plantarum. The strain with high productivity of protease was determined by 16S rRNA sequencing. (DOCX 14 kb) [file 12866_2018_1354_MOESM5_ESM.docx]

**16S rRNA gene sequence of *Lactobacillus plantarum***:

TGAATTCGAGCTCGGTACCCGGGGATCCTCTAGAGATTGTGATCCAGCCGCAGGTTCTCCTACGGCTACCTTGTTACGACTTCACCCTGATCATCTGTCCCACCTTAGGCGGCTGGTTCCTAAAAGGTTACCCCACCGACTTTGGGTGTTACAAACTCTCATGGTGTGACGGGCGGTGTGTACAAGGCCCGGGAACGTATTCACCGCGGCATGCTGATCCGGGATTACTAGCGATTCCGACTTCATGTAGGCGAGTTGCAGCCTACAATCCGAACTGAGAATGGCTTTAAGAGATTAGCTTACTCTCGCGAGTTCGCAACTCGTTGTACCATCCATTGTAGCACGTGTGTAGCCCAGGTCATAAGGGGCATGATGATTTGACGTCATCCCCACCTTCCTCCGGTTTGTCACCGGCAGTCTCACCAGAGTGCCCAACTTAATGCTGGCAACTGATAATAAGGGTTGCGCTCGTTGCGGGACTTAACCCAACATCTCACGACACGAGCTGACGACAACCATGCACCACCTGTATCCATGTCCCCGAAGGGAACGTCTAATCTCTTAGATTTGCATAGTATGTCAAGACCTGGTAAGGTTCTTCGCGTAGCTTCGAATTAAACCACATGCTCCACCGCTTGTGCGGGCCCCCGTCAATTCCTTTGAGTTTCAGCCTTGCGGCCGTACTCCCCAGGCGGAATGCTTAATGCGTTAGCTGCAGCACTGAAGGGCGGAAACCCTCCAACACTTAGCATTCATCGTTTACGGTATGGACTACCAGGGTATCTAATCCTGTTTGCTACCCATACTTTCGAGCCTCAGCGTCAGTTACAGACCAGACAGCCGCCTTCGCCACTGGTGTTCTTCCATATATCTACGCATTTCACCGCTACAATCGTCGACCTGCAGGCATGCAAGCTTGCGTAATCATGGTCATAGCTGTTTCCTGTGTGAAATTGTATCCGCTCACATCACACACATACGAGCGAGGCATAAGTGTAAAGCTGGGTGCTATGAGTGAGCTACTCACATATGCGTGCGCTCACTGCCGCTTTCAGTCGGGAAACTGTCGTGCAGCTGCAT
