# Supplementary material for: A metabolomics and proteomics study of the Lactobacillus plantarum in the grass carp fermentation
Source: BMC Microbiol. 2018 Dec 18;18:216. doi: 10.1186/s12866-018-1354-x (PMC6299570; doi:10.1186/s12866-018-1354-x)
Supplement: Supplementary file 6 — Table S1. The primer sequences for qRT-PCR. A Primer3plus software was used to design qRT-PCR analysis primers based on the genome sequence of Lactobacillus plantarum. (DOCX 15 kb) [file 12866_2018_1354_MOESM6_ESM.docx]

**Table S1.** The primer sequences for qPCR.

| Gene name | Primer sequence | Protein | Product size |
| --- | --- | --- | --- |
| 16s rRNA | F: 5′-AAGGGTTTCGGCTCGTAAAA-3′ | 16s rRNA (reference gene) | 247 bp |
|  | R: 5′-TGCACTCAAGTTTCCCAGTT-3′ |  |  |
| DnaK | F: 5′-CAACGGTCTTAACACAATCA-3′ | molecular chaperone | 220 bp |
|  | R: 5′-ATGGCTTCGGCAATCAAT-3′ |  |  |
| Pgk | F: 5′-TTCAATTCCTGATGGCTACA-3′ | phosphoglycerate kinase | 187 bp |
|  | R: 5′-GGTCGTTGCGTCTGATAA-3′ |  |  |
| RpsB | F: 5′-TCTTGACCTTGCTTACCTTC-3′ | 30S ribosomal subunit protein S2 | 215 bp |
